# Loss-of-function mutations in Zn-finger DNA-binding domain of *HNF4A* cause aberrant transcriptional regulation in liver cancer

**DOI:** 10.18632/oncotarget.25456

**Published:** 2018-05-25

**Authors:** Hiroaki Taniguchi, Akihiro Fujimoto, Hidetoshi Kono, Mayuko Furuta, Masashi Fujita, Hidewaki Nakagawa

**Affiliations:** ^1^ Laboratory for Genome Sequencing Analysis, RIKEN Center for Integrative Medical Sciences, Tokyo 108-8639, Japan; ^2^ Institute of Genetics and Animal Breeding of the Polish Academy of Sciences, Jastrzebiec 05-552, Poland; ^3^ Molecular Modeling and Simulation Group, National Institutes for Quantum and Radiological Science and Technology, Kizugawa, Kyoto 619-0215, Japan

**Keywords:** liver cancer, mutation, Zn-finger, transcription factor, HNF4A

## Abstract

Hepatocyte nuclear factors (HNF) are transcription factors that crucially regulate cell-specific gene expression in many tissues, including the liver. Of these factors, HNF4A acts both as a master regulator of liver organogenesis and a tumor suppressor in the liver. In our whole genome sequencing analysis, we found seven somatic mutations (three Zn-finger mutations, three deletion mutants, and one intron mutation) of *HNF4A* in liver cancers. Interestingly, three out of seven mutations were clustered in its Zn-finger DNA-binding domain; G79 and F83 are positioned in the DNA recognition helix and the sidechain of M125 is sticking into the core of domain. These mutations are likely to affect DNA interaction from a structural point of view. We then generated these mutants and performed *in-vitro* promoter assays as well as DNA binding assays. These three mutations reduced HNF4 transcriptional activity at promoter sites of HNF4A-target genes. Expectedly, this decrease in transcriptional activity was associated with a change in DNA binding. RNA-Seq analysis observed a strong correlation between *HNF4A* expression and expression of its target genes, *ApoB* and *HNF1A*, in liver cancers. Since knockdown of HNF4A caused a reduction in *ApoB* and *HNF1A* expression, possibly loss of HNF4 reduces the expression of these genes and subsequently tumor growth is triggered. Therefore, we propose that HNF4A mutations G79C, F83C, and M125I are functional mutations found in liver cancers and that loss of HNF4A function, through its mutation, leads to a reduction in *HNF1A* and *ApoB* gene expression with a concomitant increased risk of liver tumorigenesis.

## INTRODUCTION

Chronic liver damage arising from excessive tobacco or alcohol consumption as well as hepatitis B and C viral infections often sets the stage for the oncogenic transformation of liver cells [1–4]. Nonetheless, precise mechanism driving the oncogenic transformation of liver cells remains unclear. The development of next generation sequencing (NGS) has led to an ever-increasing number of research projects investigating the liver cancer genome. These studies have identified many single nucleotide variants and copy number alterations implicated in liver cancer [5–7]. In this regard, the International Cancer Genome Consortium (ICGC) has coordinated a large number of research projects that have the common aim of comprehensively elucidating the genomic changes present in many forms of cancers including liver cancer [5, 7–10]. Though of great interest to the scientific community, the results of these high-throughput research projects must be validated through functional studies to interpret these mutations and to expand their full potential to benefit society.

In our NGS analysis of virus-related liver cancers, the most significantly mutated genes were *CNNTB1*, *TP53*, *ARID1A/ARID2*, and *TERT* [7]. In addition to these major driver genes, we identified many low-frequency driver genes such as *HNF4A*. HNF4A is a transcription factor which plays a major role in a variety of developmental events, especially in hepatic organogenesis [11–13]. HNF4A is also a master regulator of liver-specific gene expression, and these target genes are involved in intermediary metabolism, xenobiotic and drug metabolism, and liver cancer [14–20]. HNF4A binds to a specific DNA consensus element, which is known as the DR1 binding site (AGGTCAxAGGTCA), as well as a recently identified HNF4A-specific binding element xxxxCAAAGTCCA [19]. Tissue-specific Hnf4a knock-out (KO) mice revealed that the loss of Hnf4a caused severe hepatomegaly and steatosis with a selective disruption of very-low-density lipoprotein secretion due to decreased expression of genes encoding apolipoprotein B [20, 21]. Moreover, the KO of Hnf4a suggested that Hnf4a could act as tumor suppressor in liver [21]. Interestingly, several studies demonstrated that a reduction in HNF4A activity is highly related to tumor establishment [21–23]. IDH-mutant mice, exhibiting a reduction in Hnf4a expression, expressed severe HCC liver phenotypes [18]. A recent study of Yap KO and Mst1/2 double KO mice also demonstrated severe HCC phenotypes with a concomitant reduction in Hnf4a expression [17], suggesting that the inhibition of mouse Hnf4a activity is an important trigger in tumor formation and/or progression in liver. On the other hand, germline *HNF4A* mutations are linked to maturity-onset diabetes of the young (MODY)-1, which is an atypical form of type 2 diabetes (T2D) [24, 25]. Therefore, the relationship between *HNF4A* and T2D has been extensively studied [26]. Although previous studies of Bonzo *et al*. and others have further identified HNF4A as potential tumor suppressor [21–23], the functional importance of its mutation is not well understood. Our analysis successfully revealed relatively rare gene mutations which play crucial roles in hepatic tumor formation. HNF4A controls the expression of many genes, especially those involved in metabolism and liver cell fate decisions through the binding to its target gene promoter regions via the Zn-finger DNA binding domain [27]. Thus, we here focused on the functional analysis of *HNF4A* mutations in liver cancers, namely HNF4A G79C, F83C, and M125I, which are in the Zn-finger DNA binding domain of HNF4A.

## RESULTS

### Somatic mutations of *HNF4A* in liver cancers

**Figure 1:**
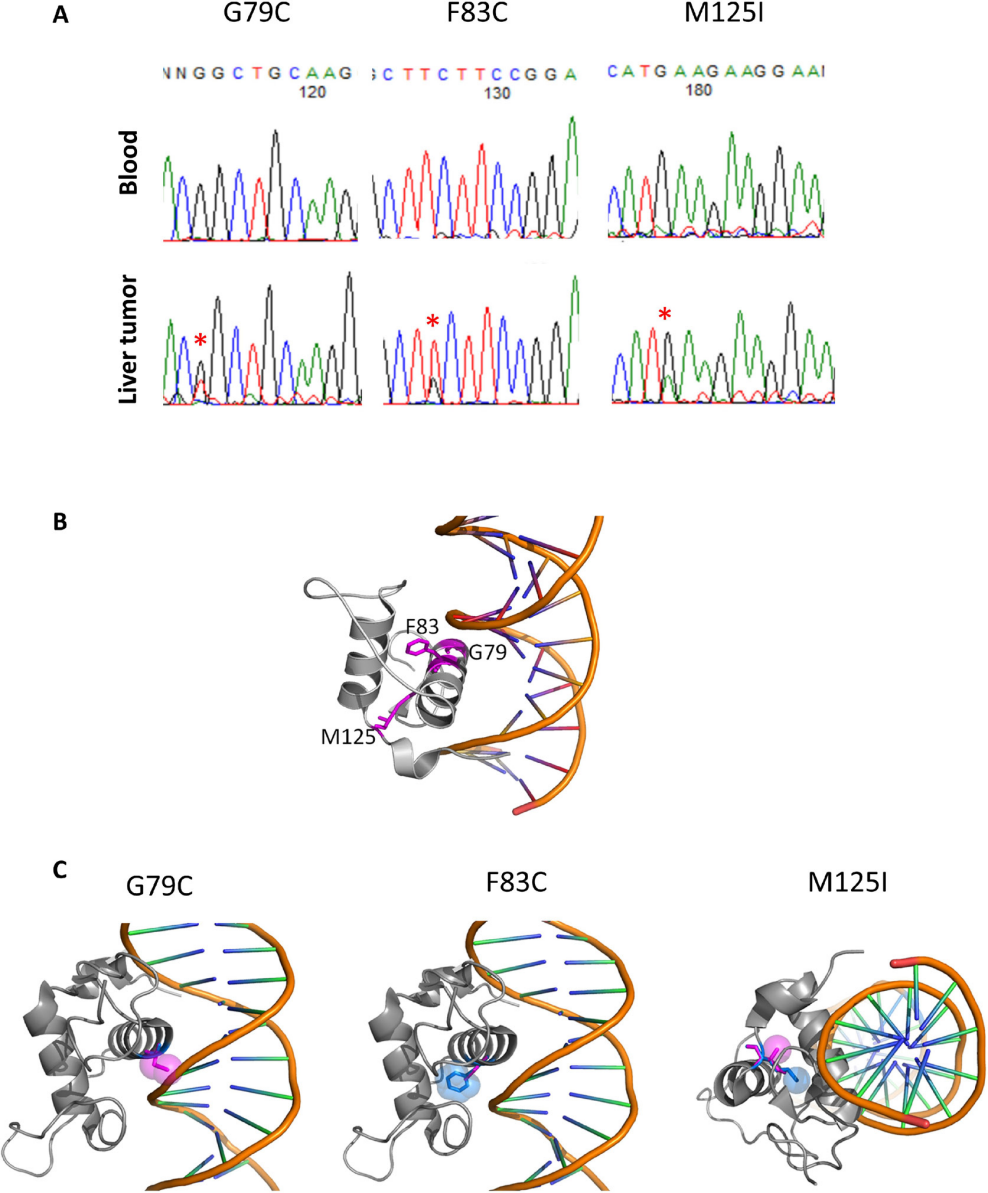
HNF4A mutations in liver cancer and their 3D structures (**A**) Detection of HNF4A mutations by Sanger sequencing in blood and liver cancer. (**B**) The locations of G79, F83 and M125 in a complex structure of the wild-type of HNF4A and DNA (PDB code: 3cbb). These amino acids are represented with stick model and are colored in magenta with residue number. The figures were drawn using pymol (The PyMOL Molecular Graphics System, Schrödinger, LLC.). (**C**) 3D-stuructures of HNF4A mutants (G79C, F83C, and M125I). The wild-type and mutated residues are colored in blue and magenta, respectively, and represented with stick and space filling models.

Our whole genome sequencing analysis of liver cancers identified *HNF4A* as a driver gene candidate [7]. Sanger sequencing validated the somatic mutations located in the Zn-finger region of *HNF4A* (G79C, F83C, and M125I, in Figure 1A). Since G79 and F83 are located in the DNA recognition helix and M125 is in the backside of the DNA recognition helix, mutation at these sites is expected to have a large impact on DNA-binding and protein stability (Figure 1B). To examine effect of the mutations from a structural point of view, we modelled 3D-structures of the mutants based on a crystal structure (PDB ID: 3cbb) by replacing a sidechain with the structurally best fit rotamer from a rotamer library using pymol software (The PyMOL Molecular Graphics System, Version 2.0 Schrödinger, LLC). In case of G79C, any rotamer did not fit in at the position and the mutation caused a clash with the phosphate backbone of DNA. F83C mutant produced a cavity in the interior of the protein, which surely destabilizes the protein. M125I also produced a cavity, destabilizing the protein (Figure 1C).

**Figure 2:**
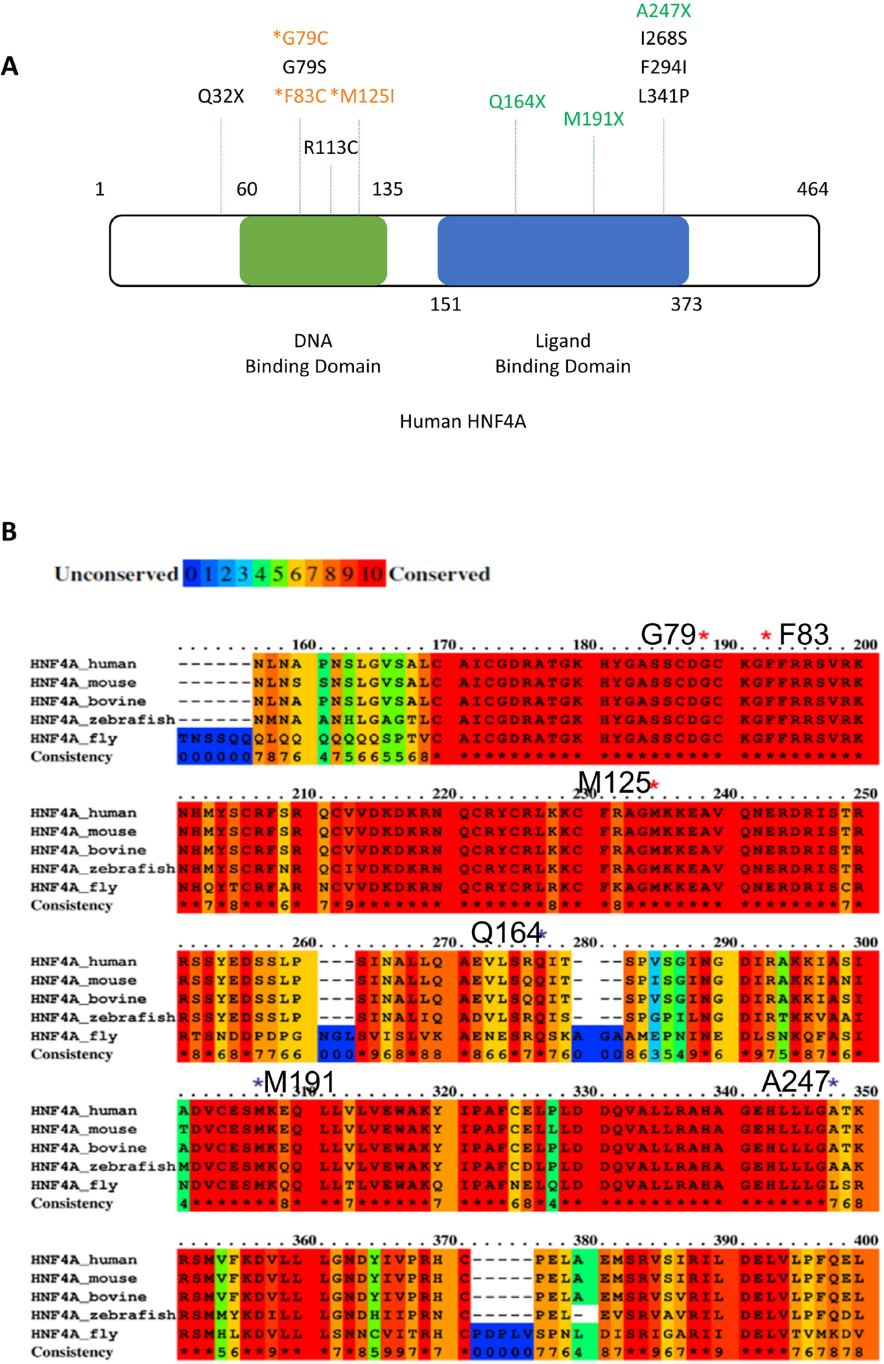
Positions of *HNF4A* mutations in liver cancer (**A**) Positions of novel mutations are indicated in the human HNF4A protein structure (DNA binding domain; green, Ligand biding domain; blue). (**B**) The alignment of the human, mouse, bovine, zebrafish, and fly HNF4A amino acid sequence and mutations found in our NGS analysis. Red color box shows highly conserved (100%) elements among the species. HNF4A mutations were indicated by asterisks.

In the ICGC database (Release 23, https://dcc.icgc.org/), there are other HNF4A mutations reported, which are mostly located in the Zn-finger region or the ligand binding domain of HNF4A (Figure 2A and Table 1). Five mutations (G79C, G79S, F83C, R113C, and M125I) in the Zn-finger region and six mutations (Q164X, M191X, A247X, I268S, F294I, L341P) in the ligand binding domain are listed in the database. These data suggest that HNF4A mutations in these regions may have some impacts on liver carcinogenesis. From an evolutionary perspective, the Zn-finger mutations found in this study are well conserved among various species including fly (Figure 2B). The asterisks in red indicate the location of the Zn-finger mutations (G79, F83, and M125) and the asterisks in black indicate the location of ligand domain mutations. The conserved domains among the analyzed species (human, mouse, bovine, zebrafish, and fly) are highlighted in red and the domains that we performed functional analysis in this study are 100% conserved throughout the different species. The mutations in such evolutionary conserved elements suggest a strong effect on the protein function although further studies are necessary.

**Table 1:** Novel human HNF4A mutations identified in 300 liver cancer patients and in ICGC database

ID	Donor ID	RIKEN ID	ICGC Project	Position	DNA change	Protein change	Consequence	Verification Status	Disease-free survival (M)
MU29810660	DO50767		LICA-FR	chr20:g.43030106	C > T	p.Q32X	pathogenic	tested and verified	
MU29810680	DO50751		LICA-FR	chr20:g.43034709	insT	Frame shift	pathogenic	tested and verified	
MU861370	DO23436		LINC-JP	chr20:g.43034732	delG	Frame shift	pathogenic	not tested	
MU31515711	DO50834	RK282	LIRI-JP	chr20:g.43034817	G > T	p.G79C	pathogenic	tested and verified	56 M free
MU29410029	DO52396		LIHM-FR	chr20:g.43034817	G > A	p.G79S	pathogenic?	not tested	
MU31043472	DO50829	RK277	LIRI-JP	chr20:g.43034830	T > G	p.F83C	pathogenic	tested and verified	59 M free
MU3451867	DO45225	RK106	LIRI-JP	chr20:g.43034873	del GTGAGGA GCCTCAATTTC	splicing donor change	pathogenic	not tested	70 M free
MU29697520	DO48483		LIHC-US	chr20:g.43036067	C > T	p.R113C		not tested	
MU3379694	DO45247	RK141	LIRI-JP	chr20:g.43036105	G > A	p.M125I	pathogenic	tested and verified	51 M free
MU3297063	DO45237	RK126	LIRI-JP	chr20:g.43042438	C > T	p.Q164X	pathogenic	tested	recurrent 56 M, survival 68 M
MU5623417	DO48732	RK200	LIRI-JP	chr20:g.43043118	del CAGCATTTT CTTCCCTGTATC TCTCGAAGA	frameshift	pathogenic	not tested	recurrent 12 M, survival 55 M
MU2808102	DO45096	RK006	LIRI-JP	chr20:g.43047101	del CT	frameshift	pathogenic	tested	recurrent 12 M, death 43 M
MU29810713	DO50952		LICA-FR	chr20:g.43048427	T > G	p.I268S		not tested	
MU29810719	DO44864		LICA-FR	chr20:g.43048504	T > A	p.F294I		not tested	
MU845816	DO23122		LINC-JP	chr20:g.43052787	T > C	p.L341P		not tested	

### *HNF4A* mutants display reduced transcriptional activity

**Figure 3:**
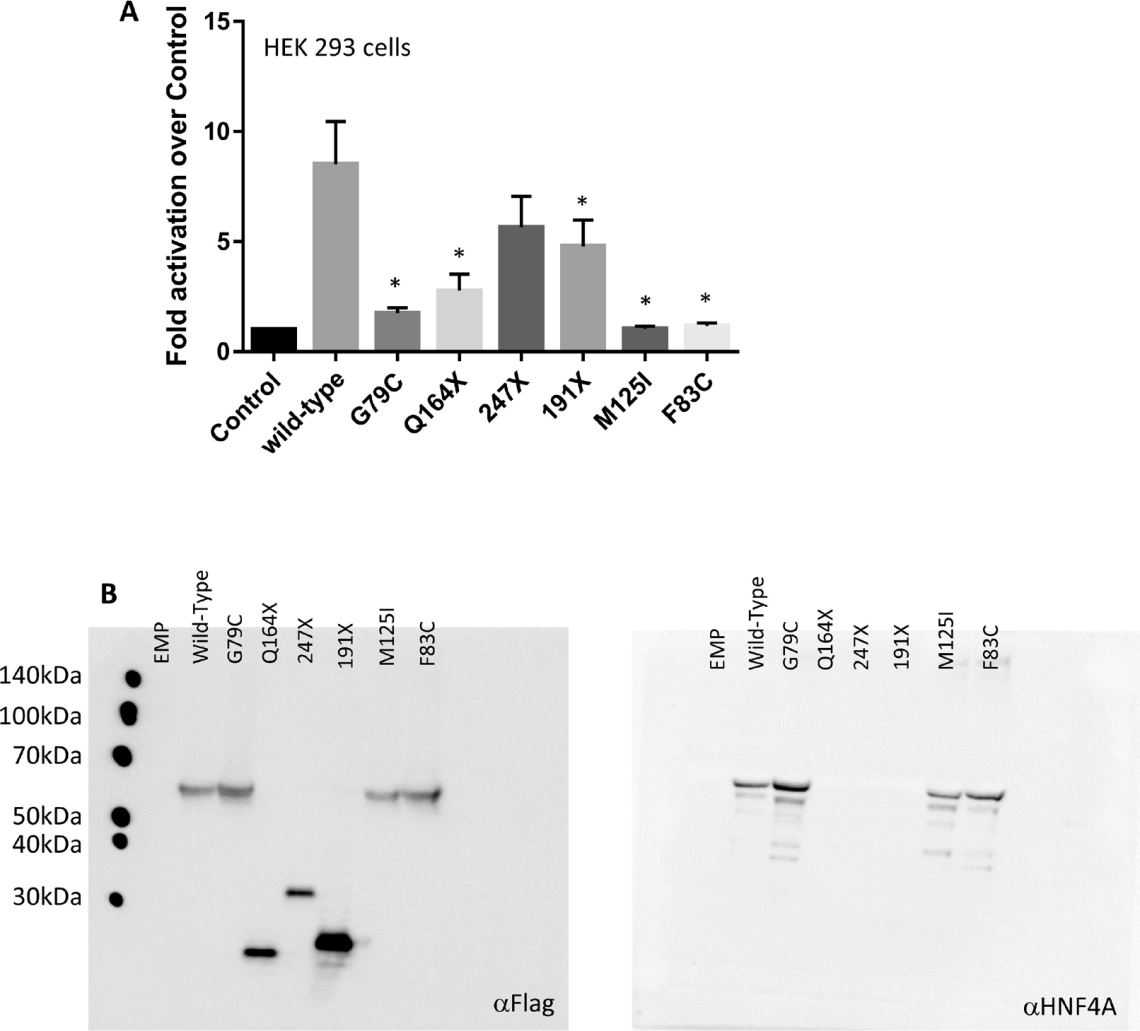
*HNF4A* mutations affect its transcriptional activity at target promoter regions (**A**) The ability of the wild-type (WT) and mutant HNF4A to transactivate target promoters when overexpressed in HEK293. The cells were co-transfected with the indicated luciferase reporters along with either an empty expression vector (serving as a control) or expression vectors (50 ng) for the indicated HNF4A proteins in 24-well culture plates. The bars indicate fold activation of HNF4A WT and mutants (vs control) on HNF4A target promoter. The corresponding promoter activity is reported as fold activation over control (±SD, *n* = 3). We performed there independent experiment and each experiment was performed as duplicate. Data reported represent the average of three experiments, each done in duplicate. Statistical significance between WT and mutants (G79C, F83C, M125I, Q164X, and 191X) is indicated as asterisks: *p*-value < 0.05 (Student's *t*-test) (**B**) HEK293 cells were transfected with expression vectors encoding WT HNF4A or the indicated mutants. Western blot analysis shows that all proteins were properly expressed.

To investigate the effect of these human *HNF4A* mutations in the Zn-finger regions, we examined the transcriptional activity of three missense mutants (G79C, F83C, and M125I), which were detected in our report [7]. The *HNF4A* mutants were first compared with the wild-type protein for their ability to trans-activate HNF4A-responsive element containing promoters. These experiments were conducted in heterologous HEK 293 cells lacking endogenous HNF4A expression (Figure 3A). Although the wild-type HNF4A overexpression induced the activity of the HNF4A-responsive element containing promoters, the HNF4A G79C, F83C, and M125I mutations completely lost their transcriptional activity.

Western blot analysis was performed to assess the overexpression of the Flag-tagged HNF4A wild-type and the Zn-finger mutants. The result shows that the HNF4A wild-type and the Zn-finger mutants are equally expressed and they are also detected by the HNF4A antibody. Thus, it is concluded that the observed effect was intrinsic to the mutant proteins since neither the expression level nor the cellular localization of the overexpressed proteins were affected (Figure 3B). Similar results were also observed in HuH7 cells (data not shown). Furthermore, our functional analysis reveals that the mutations in the ligand binding domain caused a reduction of the HNF4A transcriptional activity.

Much like the Zn-finger domain, the HNF4A ligand binding domain is important to exert the proper transcriptional activity. Therefore, it is possible that mutations in the ligand binding domain cause a defect in the transcriptional activity of HNF4A. Indeed, other HNF4A mutants (Q164X, 191X), which lead to truncation of the ligand binding domain, also reduced their transcriptional activity (Figure 3A), suggesting that the mutations located in the ligand binding domain of HNF4A merit further study. Interestingly, ICGC database has also identified five potential *HNF4A* mutations in the Zn-finger region (G79S) and the ligand binding domain (I268S, F294I, and L341P) of HNF4A, as well as one mutation (Q32X) in the N-terminal region of HNF4A (Figure 2A), suggesting that these mutations are possibly functional in liver cancer development. Further studies are needed to clarify this point.

### HNF4A mutants have reduced DNA-binding affinity

**Figure 4:**
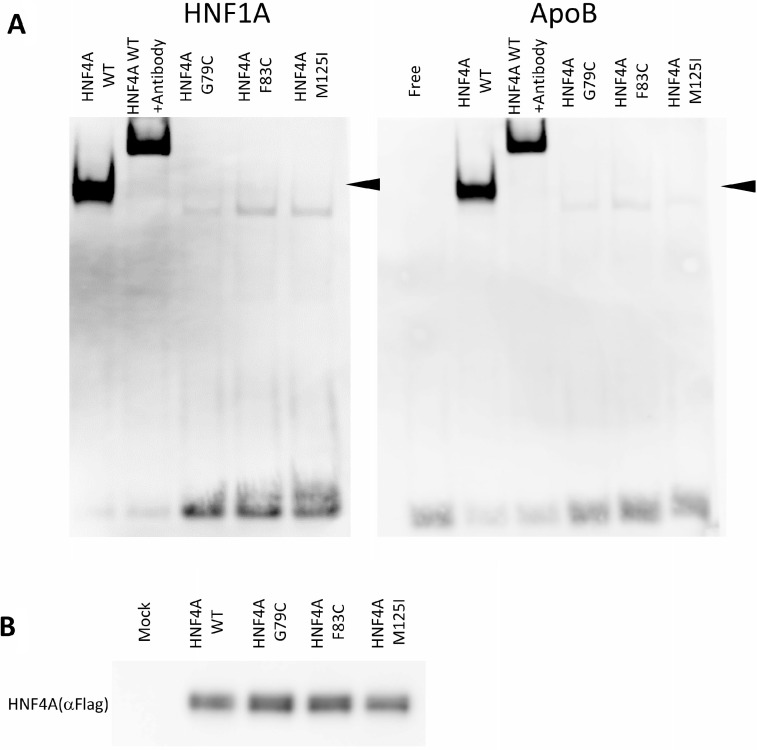
DNA-binding properties of HNF4A mutants (**A**) Electrophoretic mobility shift assays were used to assess the binding of WT or mutated HNF4A nuclear proteins to a double-stranded oligonucleotide corresponding to the consensus HNF4A binding elements of the *HNF1A* and *ApoB* promoter regions. For all the experiments, HNF4A binding was supershifted using a HNF4A antiserum. (**B**) HEK293 cells were transfected with expression vectors encoding WT HNF4A or the indicated mutants. Western blot analysis shows that all proteins were similarly expressed.

The reduced transcriptional activity of certain HNF4A mutants suggests that amino acid alterations may directly influence HNF4A DNA binding. In this regard, we compared the ability of the wild-type and mutant HNF4A proteins to bind to the HNF4A binding elements of *HNF1A* and *Apolipoprotein B* (*ApoB*) promoters using EMSA analysis. Along with reduced transcriptional activity, HNF4A G79C, F83C, and M125I mutants show a markedly reduced binding to *HNF1A* and *ApoB* promoters compared to the wild-type HNF4A (Figure 4A), though expression levels of the wild-type and mutant HNF4A proteins were similar (Figure 4B). Moreover, the fact that the HNF4A antibody shifted the binding of HNF4A to the *HNF1A* and *ApoB* promoter DNA sequences suggests that the binding found in this study is specific for HNF4A/DNA binding.

**Figure 5:**
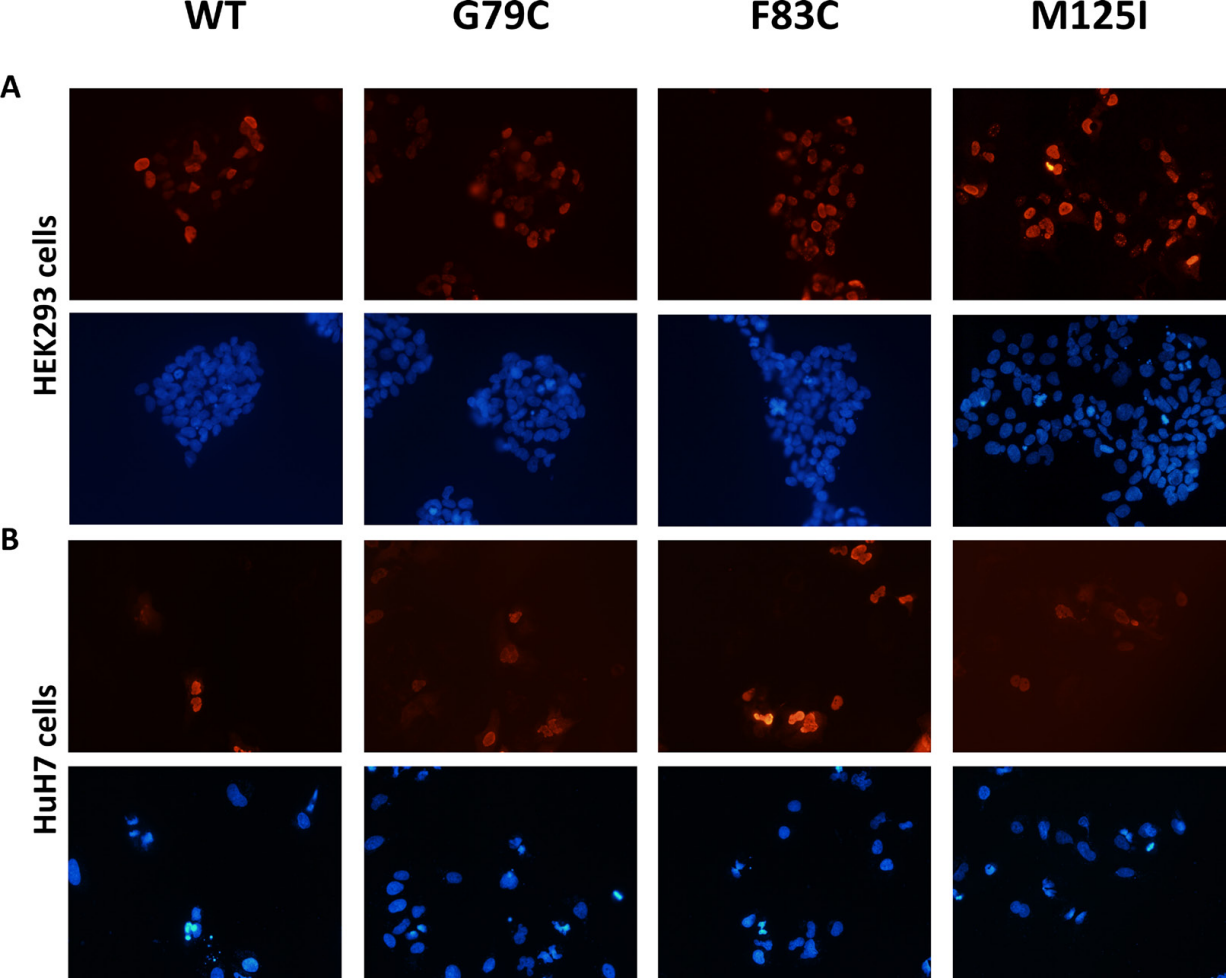
Nuclear localization of HNF4A mutants using immunofluorescent staining Cellular localization of WT and mutant HNF4A was visualized in (**A**) HEK293 cells and in (**B**) HuH7 cells using immunofluorescent staining. The nuclei were stained with DAPI and the images were taken at 10× magnification.

Since the nuclear localization may alter HNF4A mutant transcriptional activity, we have tested whether HNF4A mutants have proper nuclear localization ability. The localization of these mutant proteins did not change in HEK293 or HuH7 cells. Immunofluorescent staining shows that both the wild-type and mutants HNF4A were localized in the nucleus of HEK293 and HuH7 cells (Figure 5). These results together with the loss of transcriptional activity caused by the mutations suggest that HNF4A G79C, F83C, and M125I mutants have reduced transcriptional activity due to the loss of their ability to bind to target elements of promoter regions and not because of changes in their nuclear localization. Since the nuclear localization signal of HNF4A is located at the C-terminus, the Zn-finger mutations did not appear to disrupt nuclear localization signal. This is supported by the result that the wild-type and mutant HNF4A proteins were similarly expressed in the nucleus (Figure 4B).

### HNF4A expression is silenced in undifferentiated liver cancer cells

**Figure 6:**
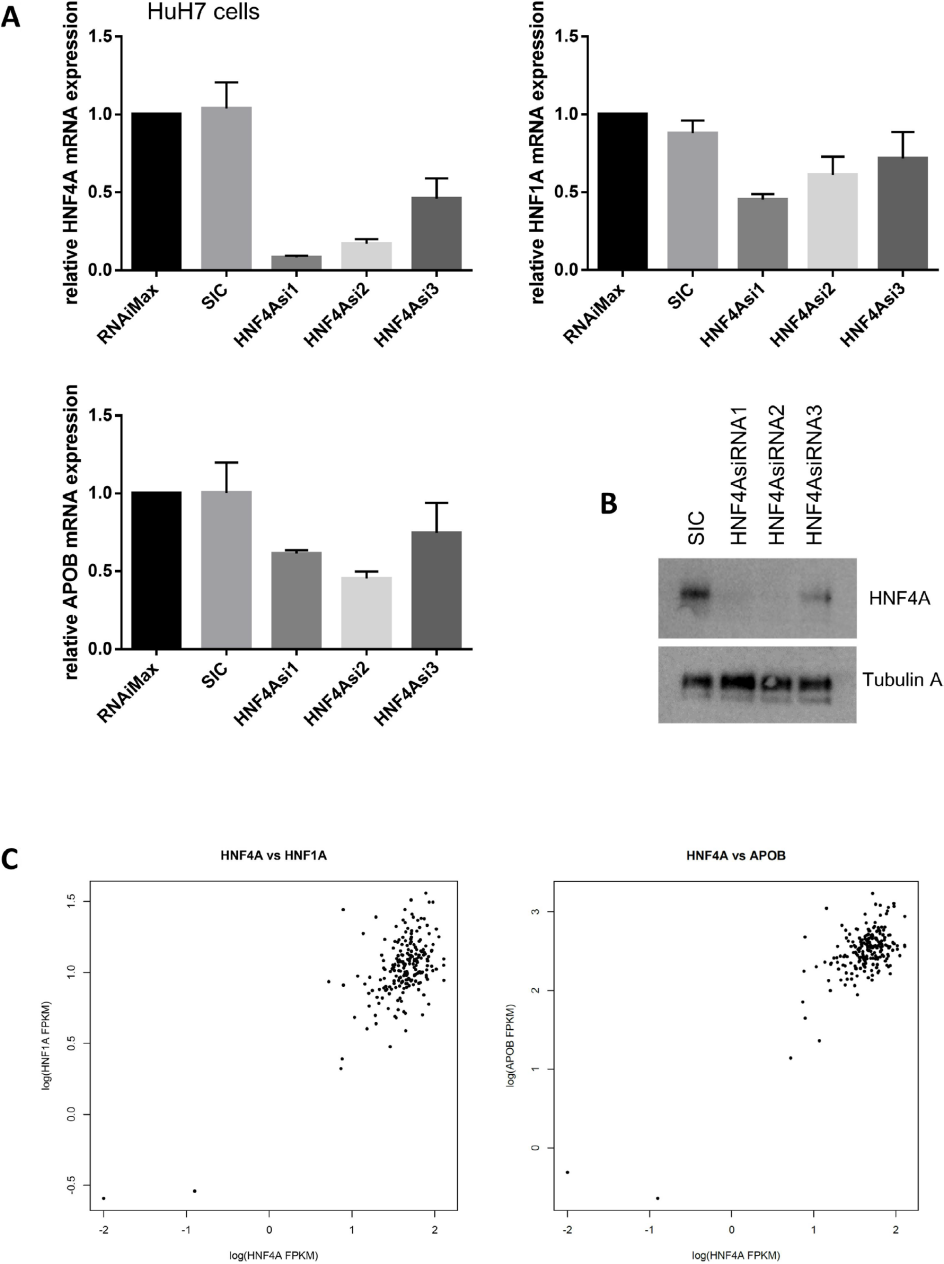
*HNF4A* expression is highly correlated with *HNF1A* and *APOB* expression (**A**) HNF4A modulates *HNF1A* and *APOB* mRNA expression. HuH7 cells were transfected with either no treatment (RNAiMax: Lipofectamine^®^ RNAiMAX), 20 nM of control siRNA (SIC; MISSION^®^ siRNA Universal Negative Control #1) or HNF4A-specific siRNAs. The results were quantitated and then normalized using *GAPDH* expression. (**B**) Western blot analysis shows that HNF4A expression was decreased in HNF4A siRNAs-treated HuH7 cells. (**C**) RNA sequencing revealed that *HNF4A* mRNA expression is highly correlated with *HNF1A* and *APOB* mRNA expression. We analyzed RNA-seq of 210 hepatocellular carcinoma (HCC), which was a subset of previously published data candidate [7]. *HNF4A* vs. *HNF1A*; correlation coefficient = 0.6813578, *p*-value < 2.2 × 10^−16^ and *HNF4A* vs. *APOB*; correlation coefficient = 0.7737859, *p*-value < 2.2 × 10^−16^

Our siRNA-mediated knockdown of HNF4A led to a significant reduction in *HNF1A* and *APOB* mRNA expression in HuH7 cells (Figure 6A and 6B). We have used three different siRNAs for *HNF4A* and all the siRNAs showed similar results whereas control siRNA did not show any effect on the *HNF1A* and *APOB* expressions. Similarly, HNF1A protein reduction was found when HuH7 cells were treated with siRNA for HNF4A whereas the expression of Lamin B1 was not altered by the treatment of HNF4A siRNA (Supplementary Figure 1). These results suggest that HNF4 controls *HNF1A* and *APOB* expressions. To confirm the correlation of HNA4A and its target gene expressions, we analyzed our RNA-seq data from 210 HCC patients [7].

Interestingly, our data from HuH7 cells is consistent with RNA-seq data from liver cancer patients in which the expression of *HNF4A*, *HNF1A*, and *APOB* mRNA are significantly correlated (*HNF4A* vs. *HNF1A*; correlation coefficient= 0.6813578, *p*-value < 2.2 × 10^−16^ and *HNF4A* vs. *APOB*; correlation coefficient = 0.7737859, *p*-value < 2.2 × 10^−16^, Figure 6C). Thus, similar to previous studies that demonstrate the importance of HNF4A in the regulation of *HNF1A* and *ApoB* expressions, our findings strongly suggest that HNF4A is a master regulator of liver cell differentiation and lipid metabolism and support the idea that any disruption of this mechanism may cause liver cancer development and progression.

RT-PCR and Western blot analysis revealed that high levels of *HNF4A* mRNA and HNF4A protein expression were found in highly differentiated liver cancer cells but not in undifferentiated liver cancer cells, including HLE and HLF cells (Supplementary Figure 2). Furthermore, we have re-analyzed RNA-seq data of hepatocellular carcinomas (HCC), a subset of previously published data [7], and evaluated overall survival of HCC patients. In this analysis, patients were stratified according to HNF4A expression. The “high” expression group made up the 75th percentile and the “low” expression group encompassed the 25th percentile. The overall survival rates of the two sets were compared with the log-rank test. We found that low *HNF4A* expression was associated with worse prognosis in liver cancers (*p*-value < 0.05, Supplementary Figure 3).

## DISCUSSION

Our previous study presented a systematic analysis of the distribution of mutations within 3D protein structures and further identified several genes governing oncogenesis [6]. Accordingly, HNF4A's molecular functions are highly related to its 3D protein structure [27]. A significant proportion of the HNF4A mutations was distributed near its Zn-finger DNA-binding domain (Figures 1 and 2). This implies that the HNF4A mutations we identified are pathogenic mutations. In particular, G79C and F83C mutations are both located within the DNA-recognition helix. These residues do not directly interact with DNA bases (Figure 1B). A closer structural observation suggests that mutation at these sites may change the relative position of DNA recognition helix [27, 28]. We therefore speculate that these two mutations lead to a diminished binding capacity and this in turn impairs HNF4A's transcriptional ability. The remaining mutation (M125I) may also destabilize the protein itself because side-chain of M125 is extended to the core of domain where a dense packing of residues are achieved. M125I mutation is thought to corrupt or change a hydrophobic packing, destabilizing the domain. Therefore, we infer that this mutation indirectly imparts DNA-binding affinity. Accordingly, combining our method to detect the distribution of mutations within a protein's 3D structure with the information from functional domain databases may help to identify genuine hot spot mutations with greater accuracy. Further structural and thermodynamical studies are required to fully elucidate the relevance and functional impact of these tumor mutations.

**Figure 7:**
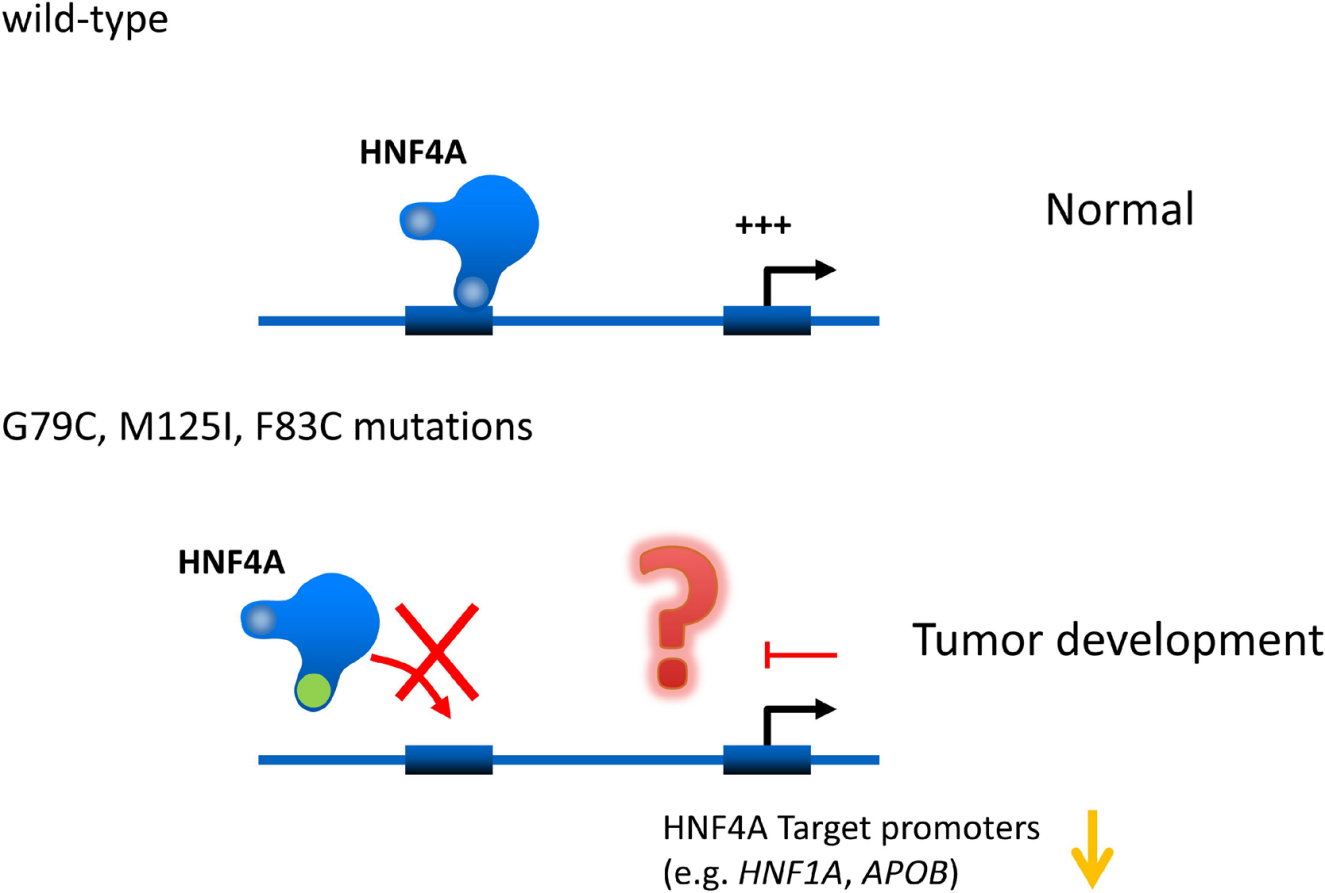
Hypothetical effects of novel human HNF4A mutations on its transcriptional activity on target promoters Novel human HNF4A mutations (G79C, F83C, M125I) lose their ability to bind to target promoters (e.g. *HNF1A* and *APOB*) leading to a reduction in transcriptional activity. This loss of function caused by HNF4A mutations may induce cell growth in liver tumors.

HNF4A mutations are well studied in MODY. The p.R76W mutation of HNF4A causes congenital hyperinsulinemia associated with Fanconi syndrome. The HNF4A M125I mutation has also been identified as the cause of hyperinsulinemic hypoglycemia [29]. However, they have not yet been reported in liver cancer patients. Our current study identifies for the first time functional HNF4A mutations in liver cancers. Interestingly, HNF4A's DNA binding ability and transcriptional activity are lost in the mutations. These results suggest that HNF4A loss-of-function mutations cause reduction of HNF4A target gene expressions and in turn this event may induce hepatic tumorigenesis and/or tumor growth (Figure 7).

We also identified three mutations located within the ligand binding domain and Q164X and 191X mutants exhibited a reduction of the transcriptional activity (Figure 3A). HNF4A's ligand domain possibly interacts with several transcription factors including GATA4 and HNF1A [30, 31]. Interestingly, the amino acids where we found mutations in HNF4A are conserved among several organisms from human to fly (Figure 2B). Therefore, it is possible that mutations at evolutionary conserved positions in HNF4A Zn-finger region and ligand domain may augment risk of triggering liver cancer development. However, these mutations reduced HNF4A transcriptional activity but did not completely abolish it. This occurs since the DNA binding domain is intact, while the ligand domain is altered, leading to diminished binding between HNF4A and its partner proteins. On the other hand, A247X is located at the C-terminal of the HNF4A ligand binding domain and this mutant maintains some portion of its transcriptional activity (Figure 3A). Accordingly, the mutant may also still possess its ability to bind to the partner proteins.

Our study presents several liver cancer-related HNF4A mutations that lead to alterations in DNA binding and/or transcription activity. Recent studies have demonstrated that HNF4A expression governs the state of liver tumor progression. In fact, genetic ablation of HNF4A in a mouse model prompted severe hepatomegaly and steatosis [20, 21]. Moreover, another study revealed that mutant IDH1 and 2, the most common genetic alterations found in intrahepatic cholangiocarcinoma, inhibit HNF4A expression, block hepatocyte differentiation and induce biliary cancer formation [18]. YAP activation has also been shown to block hepatocyte differentiation in advanced HCC. This in turn leads to tumor progression in the context of decreased *HNF4A* mRNA expression [17]. Dysregulation of *HNF4A* mRNA expression may therefore play a role in tumor development. In this regard, we identified reduced *HNF4A* mRNA and protein expressions in undifferentiated liver cancer cell lines.

Recently, two well-differentiated and non-proliferative subclasses of hepatocellular carcinoma (HCC), known as periportal-type (wild-type CTNNB1) and perivenous-type (mutant CTNNB1), have been identified. These subclasses of hepatocellular carcinoma are associated with negatively correlated gene networks [32]. Interestingly, the periportal subclass represents 29% of all HCCs and expresses a transcription factor HNF4A-driven gene network that is down-regulated in Hnf4a-KO mice [33]. This suggests that HNF4A modulates not only liver tissue differentiation but also liver cancer cell fate. Additionally, loss of HNF4A function is associated with perivenous-type HCC and poor prognosis [32]. In fact, our data demonstrates that *HNF4A* mRNA expression is lower in undifferentiated liver cancer cells (aggressive cancer cells). Additionally, we and others have identified 11 mutations (ICGC data from Japan, France, US) in the HNF4A protein sequence. Ten of these mutations are located in either the Zn- finger region or the ligand binding domain (Table 1) and may therefore be pathogenic mutations. Together, overall findings suggest that both HNF4A mutations and decreased HNF4A expression may increase the risk of hepatic tumor formation and/or progression.

## MATERIALS AND METHODS

### Cell culture

HEK293 and HuH7 liver cancer cells were cultured in Dulbecco's modified Eagle's medium (DMEM) (Gibco) that was supplemented with 10% fetal bovine serum (FBS) (Invitrogen), 4,500 mg/liter glucose, 40 μg/ml streptomycin, and 40 units/ml penicillin. The cells were stored in a humidified incubator at 5% CO_2_ and 37°C.

### Knockdown experiment by siRNA

The cells were treated with 20 nM of either control or HNF4A siRNAs and cultured for 48 h in medium without antibiotics, according to the manufacturer's instruction. The sequences of the siRNAs and primers employed in the present study are listed in Supplementary Table 1 in the Supplementary Materials.

### RNA extraction and real-time quantitative PCR (RT-qPCR)

Total RNA was extracted from cells with the RNAeasy mini kit (Qiagen), and subjected to cDNA synthesis with random primers and superscript II reverse transcriptase (Invitrogen), according to the manufacturer's protocol. RT-qPCR was performed using the KAPA SYBR FAST qPCR Master Mix (Kapa Biosystems) and a LightCycler^®^ 480 System (Roche). *GAPDH* expression was utilized for normalization.

### Western blotting

HuH7 cells were treated with different types of HNF4A siRNAs at a concentration of 20 nM for 48 h and subjected to preparation as whole-cell extracts with lysis buffer (20 mM sodium phosphate [pH 7.0], 150 mM KCl, 30 mM sodium pyrophosphate, 0.1% NP-40, 5 mM EDTA, and 1× protease inhibitor cocktail [Roche]). Cellular debris was removed by centrifugation for 5 minutes at 13000 g and 4°C. Protein concentrations were determined using the Pierce BCA Protein Assay kit. Protein samples were loaded on a 10% SDS-polyacrylamide Wako Super Sep Ace precast gel (Wako), separated, and transferred to a PVDF Membrane using the iBlot^®^ 7-Minute Blotting System (Invitorgen). Membranes were blocked with 5% skim milk and then incubated with the antibodies: anti-Flag (M2; Sigma), anti-α-tubulin (DM1A; Sigma), anti-HNF4A (3113S; CST), anti-HNF1A (Santa Cruz; F7, sc393925), and anti-Lamin B1 (Santa Cruz; C12, sc365214). The blots were treated with a horseradish peroxidase-conjugated secondary antibody (Invitrogen) and were developed using an enhanced chemiluminescence (ECL) kit (RPN2106; GE Healthcare). The signal was detected by ImageQuant LAS 4000 mini (GE Healthcare).

### Immunofluorescence

Cells were fixed by incubating them in 4% paraformaldehyde for 15 min at room temperature. After washing the cells with PBS 0.1% Tween-20 (PBST), the cells were blocked in 1% skim milk for 20 min at room temperature. Next, the cells were rinsed once with PBS 0.1% Tween-20 (PBST), treated with PBS 0.5% Tween-20 (PBST) for 10 min, incubated with mouse monoclonal FLAG-antibody followed by extensive washes and incubation with Alexa546-conjugated anti-mouse IgG antibody (Life Technologies) for 1 h. After being washed with PBST three more times, the cells were stained with DAPI and examined using a fluorescence microscope.

### Reporter assay

HEK293 cells, expressing the genes indicated in the Figure 3 legend, were lysed and the luciferase activity was measured with the PicaGene dual luciferase assay system (Toyo lnk) and ARVO (Perkin Elmer).

### EMSA

Oligonucleotides synthesized by Sigma-Aldrich were used for DNA binding assays. Sequence information is provided in the Supplementary Table 1. Double-stranded probes were generated by heating equal molar amounts of each of the 5′ to 3′ oligonucleotides with its respective complementary oligonucleotide at 95°C for 10 min, followed by cooling to room temperature. Next, double-stranded oligonucleotides were labeled with DIG-11-ddUTP using a recombinant terminal transferase (20 units/ml) in a final volume of 25 μl; according to the DIG Gel Shift Kit, Second Generation instructions (Roche Applied Science). EMSA was performed according to the manufacturer's directions. In brief, DNA binding reactions were set up using 5 μg of nuclear extract [34] of either wild-type or mutant proteins. These proteins were mixed with the above mentioned DIG-labeled oligonucleotides in a DNA binding buffer containing 1 μg of poly(dI-dC) and 0.1 μg of poly-l-lysine, in a final reaction volume of 20 μl. For supershift assays, 1 μl of HNF4A antibody (3113S; CST) was added to the nuclear proteins prior to the addition of the probe.

### RNA-seq analysis

We analyzed RNA-seq of 210 liver cancer, which was a subset of previously published data candidate [7]. Sequence reads were mapped onto GRCh37 using TopHat v2.1.1, and reads per transcript were counted using HTSeq and GENCODE v19 as a transcript annotation. The gene expression level was measured by fragments per kilobase of exon per million mapped fragments FPKM, and the correlation of gene expression between two genes was tested by the Spearman's rank correlation test. For prognosis association of *HNF4A* expression, the “high” expression group of *HNF4A* made up the 75th percentile and the “low” expression group encompassed the 25th percentile. Overall survival of informative 210 liver cancer patients was compared using the log-rank test.

### Statistical analyses

Statistical analyses were performed using the Student's *t*-test; a *p*-value < 0.05 was considered significant.

## SUPPLEMENTARY MATERIALS FIGURES AND TABLE



## References

[1] Archambeaud I, Auble H, Nahon P, Planche L, Fallot G, Faroux R, Gournay J, Samuel D, Kury S, Feray C (2015). Risk factors for hepatocellular carcinoma in Caucasian patients with non-viral cirrhosis: the importance of prior obesity. Liver Int.

[2] Donato F, Gelatti U, Limina RM, Fattovich G (2006). Southern Europe as an example of interaction between various environmental factors: a systematic review of the epidemiologic evidence. Oncogene.

[3] Gelatti U, Covolo L, Talamini R, Tagger A, Barbone F, Martelli C, Cremaschini F, Franceschi S, Ribero ML, Garte S, Nardi G, Donadon V, Donato F (2005). N-Acetyltransferase-2, glutathione S-transferase M1 and T1 genetic polymorphisms, cigarette smoking and hepatocellular carcinoma: a case-control study. Int J Cancer.

[4] Kuper H, Tzonou A, Kaklamani E, Hsieh CC, Lagiou P, Adami HO, Trichopoulos D, Stuver SO (2000). Tobacco smoking, alcohol consumption and their interaction in the causation of hepatocellular carcinoma. Int J Cancer.

[5] Alexandrov LB, Ju YS, Haase K, Van Loo P, Martincorena I, Nik-Zainal S, Totoki Y, Fujimoto A, Nakagawa H, Shibata T, Campbell PJ, Vineis P, Phillips DH (2016). Mutational signatures associated with tobacco smoking in human cancer. Science.

[6] Fujimoto A, Okada Y, Boroevich KA, Tsunoda T, Taniguchi H, Nakagawa H (2016). Systematic analysis of mutation distribution in three dimensional protein structures identifies cancer driver genes. Sci Rep.

[7] Fujimoto A, Furuta M, Totoki Y, Tsunoda T, Kato M, Shiraishi Y, Tanaka H, Taniguchi H, Kawakami Y, Ueno M, Gotoh K, Ariizumi S, Wardell CP (2016). Whole-genome mutational landscape and characterization of noncoding and structural mutations in liver cancer. Nat Genet.

[8] Cooper CS, Eeles R, Wedge DC, Van Loo P, Gundem G, Alexandrov LB, Kremeyer B, Butler A, Lynch AG, Camacho N, Massie CE, Kay J, Luxton HJ (2015). Analysis of the genetic phylogeny of multifocal prostate cancer identifies multiple independent clonal expansions in neoplastic and morphologically normal prostate tissue. Nat Genet.

[9] Alexandrov LB, Nik-Zainal S, Wedge DC, Aparicio SA, Behjati S, Biankin AV, Bignell GR, Bolli N, Borg A, Børresen-Dale AL, Boyault S, Burkhardt B, Butler AP, Australian Pancreatic Cancer Genome Initiative, ICGC Breast Cancer Consortium, ICGC MMML-Seq Consortium, ICGC PedBrain (2013). Signatures of mutational processes in human cancer. Nature.

[10] Jones DT, Hutter B, Jäger N, Korshunov A, Kool M, Warnatz HJ, Zichner T, Lambert SR, Ryzhova M, Quang DA, Fontebasso AM, Stütz AM, Hutter S, International Cancer Genome Consortium PedBrain Tumor Project (2013). Recurrent somatic alterations of FGFR1 and NTRK2 in pilocytic astrocytoma. Nat Genet.

[11] Weiss MC, Le Garrec JF, Coqueran S, Strick-Marchand H, Buckingham M (2016). Progressive developmental restriction, acquisition of left-right identity and cell growth behavior during lobe formation in mouse liver development. Development.

[12] DeLaForest A, Nagaoka M, Si-Tayeb K, Noto FK, Konopka G, Battle MA, Duncan SA (2011). HNF4A is essential for specification of hepatic progenitors from human pluripotent stem cells. Development.

[13] Parviz F, Matullo C, Garrison WD, Savatski L, Adamson JW, Ning G, Kaestner KH, Rossi JM, Zaret KS, Duncan SA (2003). Hepatocyte nuclear factor 4alpha controls the development of a hepatic epithelium and liver morphogenesis. Nat Genet.

[14] Laver TW, Colclough K, Shepherd M, Patel K, Houghton JA, Dusatkova P, Pruhova S, Morris AD, Palmer CN, McCarthy MI, Ellard S, Hattersley AT, Weedon MN (2016). The Common p.R114W HNF4A Mutation Causes a Distinct Clinical Subtype of Monogenic Diabetes. Diabetes.

[15] Barry WE, Thummel CS (2016). The Drosophila HNF4 nuclear receptor promotes glucose-stimulated insulin secretion and mitochondrial function in adults. eLife.

[16] Xu Y, Zalzala M, Xu J, Li Y, Yin L, Zhang Y (2015). A metabolic stress-inducible miR-34a-HNF4alpha pathway regulates lipid and lipoprotein metabolism. Nat Commun.

[17] Fitamant J, Kottakis F, Benhamouche S, Tian HS, Chuvin N, Parachoniak CA, Nagle JM, Perera RM, Lapouge M, Deshpande V, Zhu AX, Lai A, Min B (2015). YAP Inhibition Restores Hepatocyte Differentiation in Advanced HCC, Leading to Tumor Regression. Cell Reports.

[18] Saha SK, Parachoniak CA, Ghanta KS, Fitamant J, Ross KN, Najem MS, Gurumurthy S, Akbay EA, Sia D, Cornella H, Miltiadous O, Walesky C, Deshpande V (2014). Mutant IDH inhibits HNF-4alpha to block hepatocyte differentiation and promote biliary cancer. Nature.

[19] Fang B, Mane-Padros D, Bolotin E, Jiang T, Sladek FM (2012). Identification of a binding motif specific to HNF4 by comparative analysis of multiple nuclear receptors. Nucleic Acids Res.

[20] Hayhurst GP, Lee YH, Lambert G, Ward JM, Gonzalez FJ (2001). Hepatocyte nuclear factor 4alpha (nuclear receptor 2A1) is essential for maintenance of hepatic gene expression and lipid homeostasis. Mol Cell Biol.

[21] Bonzo JA, Ferry CH, Matsubara T, Kim JH, Gonzalez FJ (2012). Suppression of hepatocyte proliferation by hepatocyte nuclear factor 4alpha in adult mice. J Biol Chem.

[22] Lazarevich NL, Shavochkina DA, Fleishman DI, Kustova IF, Morozova OV, Chuchuev ES, Patyutko YI (2010). Deregulation of hepatocyte nuclear factor 4 (HNF4)as a marker of epithelial tumors progression. Exp Oncol.

[23] Ning BF, Ding J, Yin C, Zhong W, Wu K, Zeng X, Yang W, Chen YX, Zhang JP, Zhang X, Wang HY, Xie WF (2010). Hepatocyte nuclear factor 4 alpha suppresses the development of hepatocellular carcinoma. Cancer Res.

[24] Delvecchio M, Di Paola R, Mangiacotti D, Sacco M, Menzaghi C, Trischitta V (2014). Clinical heterogeneity of abnormal glucose homeostasis associated with the HNF4A R311H mutation. Ital J Pediatr.

[25] Stanescu DE, Hughes N, Kaplan B, Stanley CA, De Leon DD (2012). Novel presentations of congenital hyperinsulinism due to mutations in the MODY genes: HNF1A and HNF4A. J Clin Endocrinol.

[26] Saxena R, Saleheen D, Been LF, Garavito ML, Braun T, Bjonnes A, Young R, Ho WK, Rasheed A, Frossard P, Sim X, Hassanali N, Radha V (2013). Genome-wide association study identifies a novel locus contributing to type 2 diabetes susceptibility in Sikhs of Punjabi origin from India. Diabetes.

[27] Chandra V, Huang P, Potluri N, Wu D, Kim Y, Rastinejad F (2013). Multidomain integration in the structure of the HNF-4alpha nuclear receptor complex. Nature.

[28] Lu P, Rha GB, Melikishvili M, Wu G, Adkins BC, Fried MG, Chi YI (2008). Structural basis of natural promoter recognition by a unique nuclear receptor, HNF4alpha. Diabetes gene product. J Biol Chem.

[29] Arya VB, Rahman S, Senniappan S, Flanagan SE, Ellard S, Hussain K (2014). HNF4A mutation: switch from hyperinsulinaemic hypoglycaemia to maturity-onset diabetes of the young, and incretin response. Diabet Med.

[30] Song G, Pacher M, Balakrishnan A, Yuan Q, Tsay HC, Yang D, Reetz J, Brandes S, Dai Z, Putzer BM, Arauzo-Bravo MJ, Steinemann D, Luedde T (2016). Direct Reprogramming of Hepatic Myofibroblasts into Hepatocytes In Vivo Attenuates Liver Fibrosis. Cell Stem Cell.

[31] San Roman AK, Aronson BE, Krasinski SD, Shivdasani RA, Verzi MP (2015). Transcription factors GATA4 and HNF4A control distinct aspects of intestinal homeostasis in conjunction with transcription factor CDX2. J Biol Chem.

[32] Desert R, Rohart F, Canal F, Sicard M, Desille M, Renaud S, Turlin B, Bellaud P, Perret C, Clement B, Le Cao KA, Musso O (2017). Human Hepatocellular Carcinomas with a Periportal Phenotype Have the Lowest Potential for Early Recurrence after Curative Resection. Hepatology.

[33] Holloway MG, Miles GD, Dombkowski AA, Waxman DJ (2008). Liver-specific hepatocyte nuclear factor-4alpha deficiency: greater impact on gene expression in male than in female mouse liver. Mol Endocrinol.

[34] Schreiber E, Matthias P, Muller MM, Schaffner W (1989). Rapid detection of octamer binding proteins with ‘mini-extracts’, prepared from a small number of cells. Nucleic Acids Res.

