# Supplementary material for: Loss-of-function mutations in Zn-finger DNA-binding domain of HNF4A cause aberrant transcriptional regulation in liver cancer
Source: Oncotarget. 2018 May 25;9(40):26144–56. doi: 10.18632/oncotarget.25456 (PMC5995239; doi:10.18632/oncotarget.25456)
Supplement: Supplementary file 1 [file oncotarget-09-26144-s001.pdf]

Loss-of-function mutations in Zn-finger DNA-binding domain of *HNF4A* cause aberrant transcriptional regulation in liver cancer

SUPPLEMENTARY MATERIALS

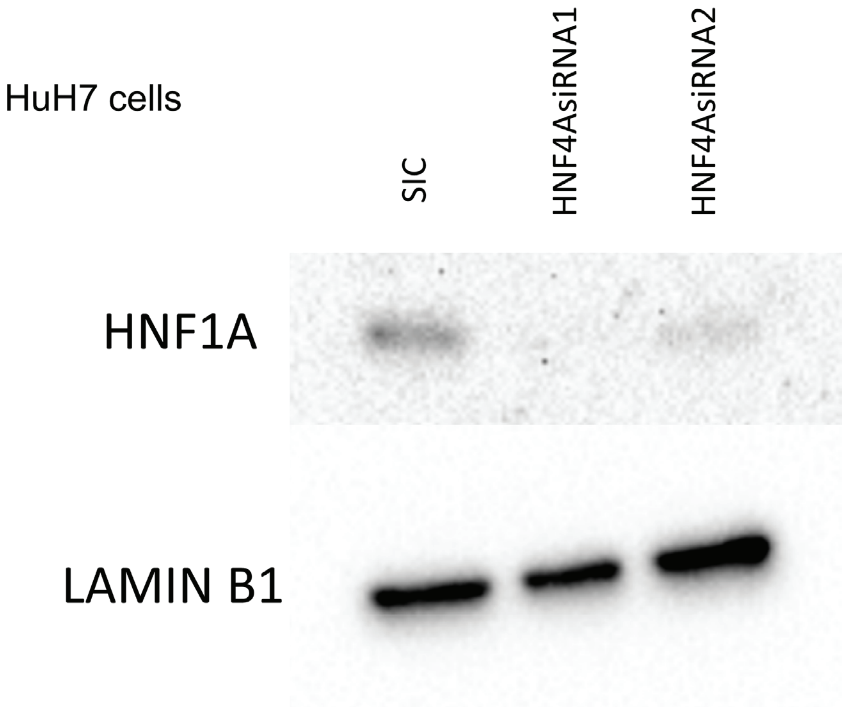

Supplementary Figure 1: HNF4A modulates HNF1A protein expression. HuH7 cells were transfected with either 20 nM of control siRNA or two HNF4A-specific siRNAs. Western blot analysis using nuclear extraction shows that HNF1A expression was decreased in HNF4A siRNAs-treated HuH7 cells. Lamin B1 was used as internal control.

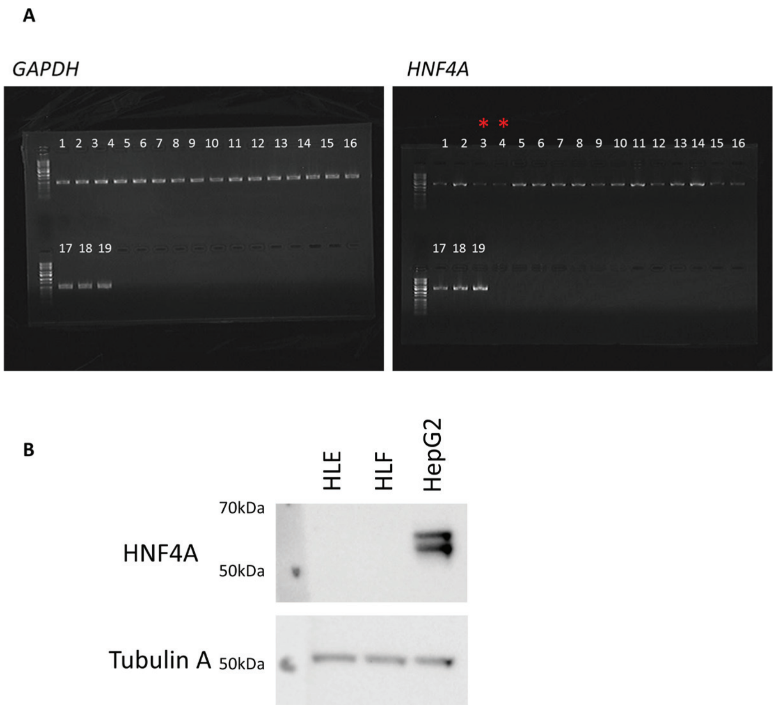

Supplementary Figure 2: Low HNF4A expressing is found in undifferentiated liver cancers. RT-PCR (**A**) (1.Alexander (differentiated hepatocellular carcinoma) 2. Hep G2 (differentiated hepatocellular carcinoma) 3. HLE (undifferentiated hepatocellular carcinoma) 4. HLF (undifferentiated hepatocellular carcinoma) 5. HuH1 6. HuH6 7. HuH7 8. JHH1 9. JHH2 10.JHH4 11.JHH5 (established from small liver cancer) 12.JHH6 13.JHH7 14.KYN2 15.Li7 16.SNU398 (poorly differentiated) 17.SNU423 (poorly differentiated) 18.SNU449 (poorly differentiated) 19.SNU475(poorly differentiated). (**B**) Western bot shows undifferentiated liver cancer cells (HLE and HLF) express low level of HNF4 protein as compared to HepG2 cells.

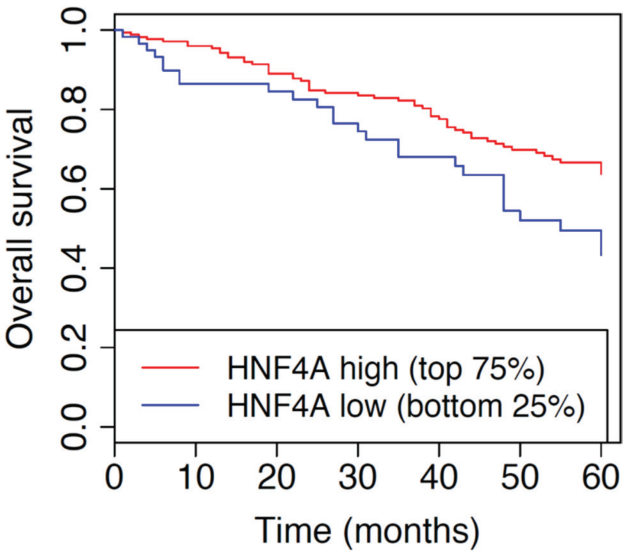

Supplementary Figure 3: The relationship between *HNF4A* expression and prognosis in liver cancer patients. The patients were stratified into two groups based on *HNF4A* expression: “high” for top 75% and “low” for bottom 25%. Their overall survival was compared using the log-rank test. Low HNF4A was associated with poor prognosis of liver cancer patients (p-value < 0.05).

Supplementary Table 1: Sequences of primers used for primers and siRNAs used in this experiment

**Oligos and siRNAs**	
hHNF4A F	TCGGTACCACGACTCTCCAAAACCCTCGT
hHNF4A R	CCTCTAGACTAGATAACTTCCTGCTTGGTGATG
hHNF4A qPCR F	GTGGTGGACAAAGACAAGAGG
hHNF4A qPCR R	CATAGCTTGACCTTCGAGTGC
hHNF1A qPCR F	AGTGAGTCCGGGCTTCACAC
hHNF1A qPCR R	TGAAGGTCTCGATGACGCTG
hAPOB qPCR F	CTCGTTACCACATGAAGGCTGA
hAPOB qPCR R	CGTAGAGACCCATCACATGATAGTG
hGAPDH qPCR F	CAAGGTCATCCATGACAACTTTG
hGAPDH qPCR R	GTCCACCACCCTGTTGCTGTAG
H4-SBM F (LUC Assay)	TCGGTACCCAAAGTCCAGGGGCAAAGTCCAAGATCTGC
H4-SBM R (LUC Assay)	GCAGATCTTGGACTTTGCCCCTGGACTTTGGGTACCGA
rHNF1A EMSA F	AAGGCTGAAGTCCAAAGTTCAGTCCCTTC
rHNF1A EMSA R	GAAGGGACTGAACTTTGGACTTCAGCCTT
hAPOB EMSA F	GGAAAGGTCCAAAGGGCGCCTTG
hAPOB EMSA R	CAAGGCGCCCTTTGGACCTTTCC
siRNA HNF4A 1 S	GAC AUU CGG GCG AAG AAG AdTdT
siRNA HNF4A 1 A	UCU UCU UCG CCC GAA UGU CdGdC
siRNA HNF4A 2 S	CAC AAU GCC CAC UCA CdTdT
siRNA HNF4A 2 A	GUG AGU GGG CAU UGU GdTdT
siRNA HNF4A 3 S	CAC AAA ACA AAG UUU ACU UdTdT
siRNA HNF4A 3 A	AAG UAA ACU UUG UUU UGU GdTdT
